# Mitochondrial and Chromosomal Damage Induced by Oxidative Stress in Zn^2+^ Ions, ZnO-Bulk and ZnO-NPs treated *Allium cepa* roots

**DOI:** 10.1038/srep40685

**Published:** 2017-01-25

**Authors:** Bilal Ahmed, Sourabh Dwivedi, Malik Zainul Abdin, Ameer Azam, Majed Al-Shaeri, Mohammad Saghir Khan, Quaiser Saquib, Abdulaziz A. Al-Khedhairy, Javed Musarrat

**Affiliations:** 1 Department of Agricultural Microbiology, Faculty of Agricultural Sciences, Aligarh Muslim University, Aligarh, 202002 UP, India; 2 Department of Applied Physics, Aligarh Muslim University, Aligarh, 202002 UP, India; 3 Department of Biotechnology, Jamia Hamdard, New Delhi, 110062, India; 4 Department of Biological Sciences, Faculty of Science, King Abdulaziz University, Jeddah, Saudi Arabia; 5 Zoology Department, College of Science, King Saud University, P.O. Box 2455, Riyadh 11451, Saudi Arabia; 6 School of Biosciences and Biodiversity, Baba Ghulam Shah Badshah University, Rajouri, J & K, India

## Abstract

Large-scale synthesis and release of nanomaterials in environment is a growing concern for human health and ecosystem. Therefore, we have investigated the cytotoxic and genotoxic potential of zinc oxide nanoparticles (ZnO-NPs), zinc oxide bulk (ZnO-Bulk), and zinc ions (Zn^2+^) in treated roots of *Allium cepa*, under hydroponic conditions. ZnO-NPs were characterized by UV-visible, XRD, FT-IR spectroscopy and TEM analyses. Bulbs of *A. cepa* exposed to ZnO-NPs (25.5 nm) for 12 h exhibited significant decrease (23 ± 8.7%) in % mitotic index and increase in chromosomal aberrations (18 ± 7.6%), in a dose-dependent manner. Transmission electron microcopy and FT-IR data suggested surface attachment, internalization and biomolecular intervention of ZnO-NPs in root cells, respectively. The levels of TBARS and antioxidant enzymes were found to be significantly greater in treated root cells vis-à-vis untreated control. Furthermore, dose-dependent increase in ROS production and alterations in Δ*Ψm* were observed in treated roots. FT-IR analysis of root tissues demonstrated symmetric and asymmetric P=O stretching of >PO_2_^−^ at 1240 cm^−1^ and stretching of C-O ribose at 1060 cm^−1^, suggestive of nuclear damage. Overall, the results elucidated *A. cepa*, as a good model for assessment of cytotoxicity and oxidative DNA damage with ZnO-NPs and Zn^2+^ in plants.

ZnO nanomaterials have enormous applications in various fields, such as manufacturing of solar cells, sensors^1^,^2^, piezoelectric devices, light emitting diodes, semiconductors^3^, and paints^4^. Besides, they are widely used in health applications such as, sun screens, cosmetics, bio-imaging, diseases diagnostics, and cancer treatment^5^,^6^,^7^,^8^. The rationale for their extensive use is the unique and fascinating electrical, optical and mechanical properties, including absorption of solar energy, high catalytic efficiency, high photo sensitivity, stability, and high band gap (3.37 eV) with large exciton binding energy (60 MeV)^9^,^10^,^11^,^12^,^13^. Furthermore, ZnO-NPs are reported to inhibit bacterial growth due to release of zinc ions from ZnO-NPs^14^, and manifest toxicity via generation of reactive oxygen species (ROS), such as hydrogen peroxide^15^. Increased expression of general stress response gene (*dna*K) and oxidative stress genes (*ahp*C and *kat*A) following exposure to ZnO-NPs, suggested induced oxidative stress in bacteria^16^. Moreover, ZnO-NPs induced oxidative stress and apoptosis have been reported in HepG2 and MCF-7 cells^17^, and DNA damage in human lymphocytes^18^.

Lately, phytotoxicity assessment of ZnO-NPs has been envisaged as a subject of investigation, since plants at the first trophic level offer a large surface area to NPs in their habitats such as soil, air, and water^19^. Primarily, root is the region of plant system, where NPs encounter first contact, starting from adsorption at root tips, rhizodermis, lateral root junctions and other plant surfaces viz. cuticle, bark, hydathodes, and stigma^19^. Uptake of NPs may occur through micro- and nanometer in size orifices in plants, and subsequently translocated or aggregated at the cellular surfaces, plasmodesmata, and/or inside the cell^19^. Accumulation and long term persistence of ZnO-NPs in edible crop plants increases the probability to enter in human food chain^20^. Furthermore, interactions of ZnO-NPs with biological molecules present in plant cells may alter the physicochemical properties and possibly trigger signal transduction pathways. Till date, limited studies have been performed on phytotoxicity of ZnO-NPs. Kouhi *et al*. investigated the effects of ZnO-NPs (<50 nm), ZnO microparticles (MPs), and zinc ions (Zn^2+^) on some growth parameters of *Brassica napus* seedlings^20^. Ramesh *et al*. reported the effects of bulk and nano-zinc oxide on seed germination root and shoot growth, mitosis, photosynthetic pigments and total protein content in *Triticum aestivum*^21^. Bandyopadhyay *et al*. compared the phytotoxicity of ZnO-NPs, bulk ZnO, and ionic zinc (ZnCl_2_) in alfalfa plants^22^. However, to the best of our understanding no systematic study either in qualitative or quantitative terms on ZnO-NPs induced plant root tissue damage, intracellular localization, intracellular ROS generation, mitochondrial damage, and chromosomal aberrations has been reported. With this aim, we have chosen *A. cepa,* as a model, for comparative assessment of the cytotoxic and genotoxic effects of ZnO-NPs, bulk ZnO and Zn^2+^ ions. *A. cepa* bioassay has been extensively used for eco-toxicological testing, and *in situ* risk evaluation of environmental contaminants^23^. This assay has also been used for assessment of chromosomal aberrations and lipid peroxidation in different studies with various NPs such as Al_2_O_3_-NPs^24^, Ag-NPs^25^,^26^, ZnO-NPs^27^,^28^, Bismuth (III) Oxide NPs^29^, MWCNT^30^, TiO_2_^31^, Copper NPs^32^, and Zinc-NPs^33^. However, no conclusive evidence for NPs interaction with plant cell components and/or constituents, cellular damage as well as role of intracellular ROS and dissipation of mitochondrial membrane potential (*Ψm*) are reported. Also, earlier studies have reported variable observations as the NPs interaction with cell depends upon their size, shape, solubility and intracellular chemical environment^34^,^35^. Therefore, in this study, we have characterized the ZnO-NPs using UV-Vis spectroscopy, XRD, FTIR, TEM and SEM analyses, and demonstrated the (i) internalization of ZnO-NPs in *A. cepa* root tissues by FTIR, TEM and SEM, (ii) intracellular ROS production, mitochondrial and chromosomal damage induced by bulk ZnO, Zn^2+^, and ZnO-NPs in *A. cepa* root cells using DCFH-DA, and Rh-123 fluorescence probes, and TEM imaging, and (iii) proposed a plausible mechanism of ZnO-NPs interaction and cellular damage in plant tissue.

## Results

### Characterization of ZnO-NPs

**Figure 1:**
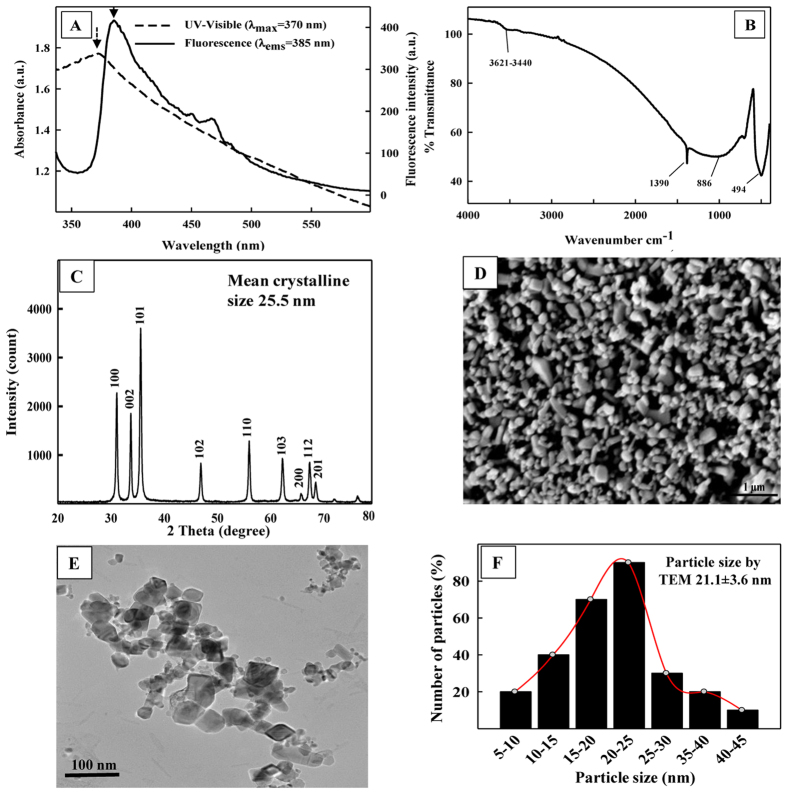
Characterization of ZnO-NPs. (**A**) UV-Visible and fluorescence spectrum of ZnO-NPs, (**B**) FTIR spectrum of ZnO-NPs, (**C**) XRD pattern of ZnO-NPs, (**D**) SEM micrograph of ZnO-NPs, (**E**) TEM micrograph of ZnO-NPs, and (**F**) frequency size distribution of ZnO-NPs by TEM.

Nanoparticles were characterized by UV-Visible, fluorescence, FT-IR spectroscopy, X-ray diffraction, TEM and SEM-EDX analyses (Fig. 1 Panels A–F). Figure 1A shows the UV-visible spectrum of ZnO-NPs, which exhibits a sharp characteristic peak at ~370 nm at room temperature indicating the purity of ZnO-NPs (Fig. 1A dashed line). Similarly, a sharp fluorescence emission peak (λ_exc_ = 320 nm) of ZnO-NPs was obtained at 385 nm (Fig. 1A solid line), which corresponds to the near band gap excitonic emission. No auxiliary band in visible region suggests the absence of oxygen vacancy in ZnO-NPs. FT-IR spectrum of ZnO-NPs shows a distinct absorption bands around wave number of 494 cm^−1^ (Fig. 1B). The results of XRD, TEM, SEM with EDX providing information regarding the shape, and size of ZnO-NPs are shown in Fig. 1C–F. XRD pattern of 2θ values of ZnO-NPs was obtained in the range of 20 to 80 (Fig. 1C). Diffraction at 31.76°, 34.46°, 36.26°, 47.57°, 56.59°, 62.92°, 66.37°, 66.97°, and 69.09° can be well indexed to 100, 002, 101, 102, 110, 103, 200, 112, and 201 Miller indices (hkl) of hexagonal ZnO wurtzite structure (JCPDS # 36-1451). Average crystallite size of ZnO-NPs (25.5 nm) was calculated based on Full-Width-at-Half-Maximum (FWHM) of 101 crystallite plane of reflection using Debye-Sherrer’s equation. The SEM image at 20,000 X, 25 kV provided the surface characteristic of ZnO-NPs, indicating small and large aggregates of variable sizes (Fig. 1D). The TEM micrograph at 40,000 X, 200 Kv revealed pleomorphic and variable size of ZnO-NPs (Fig. 1E) with an average mean particle size of 21.1 ± 3.6 (Fig. 1F).

### Optical microscopic analysis of mitosis and chromosomal aberrations

**Figure 2:**
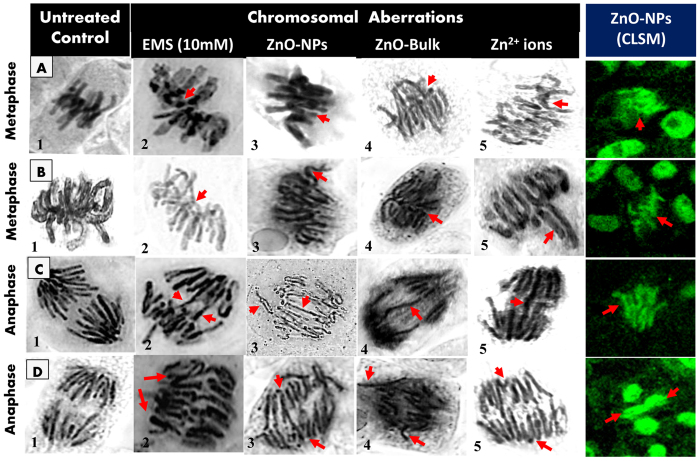
Optical and confocal microscopic analyses of chromosomal damage in *A. cepa* root meristem cells. Chromosomal aberrations induced by ZnO-NPs, ZnO-Bulk, and Zn^2+^ ions in *A. cepa* root meristem cells at different cell division stages are shown as: normal metaphase (**A1**), sticky metaphase (**A2–A5**), normal metaphase (**B1**), sticky metaphase (**B2**), sticky metaphase with loop (**B3**), sticky metaphase with laggard chromosome(**B4**), and disoriented metaphase (**B5**), normal anaphase (**C1**), chromosome bridges with lag (**C2,C3**), broken chromosome bridge (**C4**), single chromosome bridge (**C5**), normal anaphase (**D1**), sticky multipolar anaphase (**D2**), multipolar anaphase with chromosome bridges (**D3**), multipolar anaphase (**D4**), multipolar anaphase with single chromosome bridge (**D5**). EMS (10 mM) was taken as a positive control. (All images were at 1000X magnification).

Qualitative effect of ZnO-NPs, ZnO-Bulk, and Zn^2+^ ions on chromosomes at different phases of cell cycle viz. prophase, metaphase, anaphase, and telophase in untreated and treated root meristematic cells is shown in Fig. 2. Cells at each stage were scored for chromosomal aberrations (CA) such as irregular prophase, vacuolated nucleus at prophase, stickiness and disorientation at metaphase, polar deviation at anaphase, chromosome bridges with lag, multipolar anaphase and vagrant chromosomes. The cells exhibiting major chromosomal damage were quantified and genotoxicity represented in terms of mitotic index (MI) and % CAs, as a function of concentration of ZnO-NPs, ZnO-Bulk, and Zn^2+^ ions (Table 1). Results in Table 1 and Supplementary Figure S2 revealed concentration dependent reduction in MI (%) and increase in frequency of CAs (%) of treated roots compared to untreated control, respectively. The data show that the MI (%) of onion roots treated with 50, 100, 200, 500, and 1000 μg/ml was 53.06 ± 0.9, 46.46 ± 2, 42.6 ± 1.5, 37.16 ± 1.3, and 30.23 ± 0.4 for ZnO-NPs, whereas 57.4 ± 1.86, 53.4 ± 1.91, 49 ± 0.97, 44.6 ± 1.04, and 37.5 ± 2.12 for ZnO-Bulk, and 56.3 ± 2.25, 45.3 ± 4.65, 40.7 ± 1.67, 34.6 ± 2.65, and 27.7 ± 1.55 for Zn^2+^ ions. Almost similar pattern of genotoxicity was observed with the values for (%) CAs recorded as 10.4 ± 0.86, 12.03 ± 0.61, 17.16 ± 1.9, 23.4 ± 1.7, and 28.66 ± 0.87 for ZnO-NPs, 4.86 ± 1.24, 7.1 ± 1.01, 10.26 ± 1.19, 16.2 ± 1.7, and 17.33 ± 1.1 for ZnO-Bulk, 17.96 ± 1.15, 25.16 ± 0.87, 37.33 ± 1.3, 38.46 ± 0.35, and 40.5 ± 0.6 for Zn^2+^ ions, at similar concentration range under identical treatment conditions. The MI (%) for control was determined to be 58.4 ± 1.8, whereas no CAs was detected in untreated cells.

Exposure to varying concentrations (50–1000 μg/ml) of ZnO-Bulk, ZnO-NPs, and Zn^2+^, resulted in myriad of structural changes in chromosomes. An array of CAs were captured and characterized as irregular prophase, vacuolated nucleus at prophase, stickiness and disorientation at metaphase, polar deviation at anaphase, chromosome bridges with lag, multipolar anaphase and vagrant chromosomes which were very similar to those anomalies caused by EMS (10 mM) (Fig. 2). The data on chromosomal aberrations in ZnO-Bulk, ZnO-NPs, and Zn^2+^ ions treated *A. cepa* root meristem cells are shown in Table 1. Treatment of cells at 50 μg/ml concentration of ZnO-Bulk, ZnO-NPs, and Zn^2+^ ions resulted in 0.86 ± 0.35%, 2.03 ± 0.2% and 5.56 ± 0.4% chromosome stickiness, respectively, which increased significantly to 3.2 ± 1.08, 4.3 ± 0.45 and 9.93 ± 0.2% at 10-fold greater concentration of 500 μg/ml, based on scoring of 1000 cells per slide. Similarly, a concentration dependent increase in chromosome bridge formation was observed with 2.9 ± 0.1%, 4.66 ± 0.41% and 9.6 ± 0.3% bridge formation at 500 μg/ml concentration of ZnO-Bulk, ZnO-NPs, and Zn^2+^ ions, respectively. As metaphase proceeded to anaphase, sticky mass gradually became relaxed and the chromosomes that did not separate, gave rise to sticky laggards and bridges (Fig. 2).

### Confocal laser scanning microscopic (CLSM) analysis of root cells

Root tip cells of *A. cepa* grown in absence and presence of ZnO-NPs were analyzed for chromosomal aberrations under CLS microscope after staining with acridine orange (10 μg/ml). Different types of CAs such as metaphase stickiness, metaphasic disorientation, chromosome stickiness with lag, and polar disturbance at anaphase were detected (Fig. 2), which were similar to those detected in optical microscopic analysis and therefore substantiated the genotoxicity of ZnO-NPs in plant cells. Other studies have also demonstrated genotoxic effects of nanoparticles such as Al_2_O_3_-NPs^24^, and TiO_2_^31^ through confocal microscopy.

### Scanning and transmission electron microscopy of treated roots

**Figure 3:**
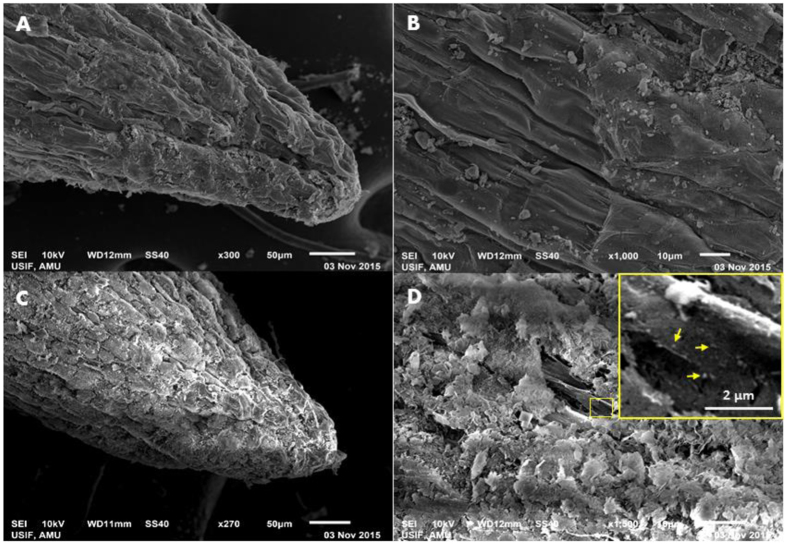
SEM micrographs showing ZnO-NPs deposition and tissue damage after exposure of *A. cepa* roots with ZnO-NPs. Panel (**A** and **B**) shows untreated control root tip and surface. Panel **C**, and **D** depict the tip and surface of ZnO-NPs (1000 μg/ml) treated roots at 50 and 10 μm scale, respectively. The attachment of nanometer scale charged particles, fissures and fractured tissues at root surface are shown by arrows (**D** inset).

Morphological changes induced by ZnO-NPs were visually observed under scanning electron microscope. The surface of untreated root was clear, smooth and intact and no charge on surface appeared during analysis (Fig. 3A,B). However, significant aberrations, nanometer scale charged particles, fissures and fractured tissues were observed on surface of roots treated with 1000 μg/ml of ZnO-NPs. Root cells were found crumbled, surface ruptured and spikes of root tissues were present throughout the root surface. In addition, the charge imposed by the aggregation of ZnO-NPs was observed during the scanning process (Fig. 3C,D). The fissures and fractures across the root tissues and nanoparticles attached to the surface are shown in Fig. 3D inset.

**Figure 4:**
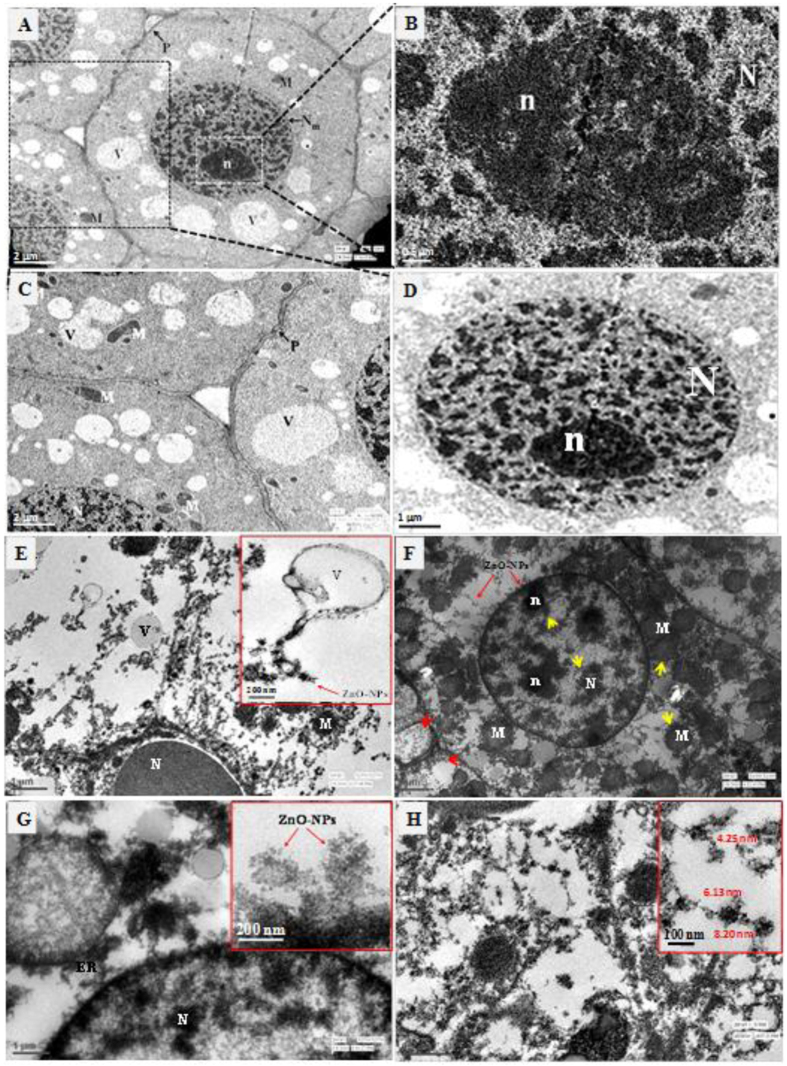
TEM micrographs showing internalization and sub-cellular damage in *A. cepa* root meristem cells upon exposure with ZnO-NPs. Untreated cell showing normal architecture of nucleus (N), nucleolus (n), integrated mitochondria (M), intact nuclear membrane (N_m_), vacuoles (V), plasmodesmata (P) (panel **A** and **B**). Triangular periplasmic space is clearly visible in untreated cell (panel **B**). Magnified view of nuclear matrix and nucleolus of untreated cell (panel **C** and **D**). Images of roots treated with ZnO-NPs (1000 μg/ml) showing the influx of ZnO-NPs in cytoplasm and attachment to the vacuoles (panel **E**). Yellow arrow heads showing degeneration of nuclear constituents and significant swelling of mitochondria while red arrows and arrow heads showing attachment of ZnO-NPs onto the nuclear membrane and infiltration of ZnO-NPs in intracellular junctions (panel **F**). Analysis was performed at 200 keV. Magnified view showing the sequesteration of ZnO-NPs on nuclear membrane (panel **G**). Distribution of ZnO-NPs in cytoplasmic matrix (panel H). Magnification for images (panel **A**) 10,000x, (panel **B**) 30,000x, (panel **C**) 12,000x, (panel **D**) 20,000x, (panel **E**) 15,000x, (panel **E** inset) 20,000x, (panel **F**) 15,000x, (panel **G**) 20,000x, (panel **G** inset) 30,000x, (panel **H**) 20,000x, and (panel **H** inset) 25,000x.

As compared to untreated control, TEM images of treated root cells showed a clear incursion and attachment of ZnO-NPs on the outer and inner surface of cell and nuclear membrane and intracellular cell junctions, plasmodesmata (Fig. 4A–H). Most likely, the ZnO-NPs could find their way inside the cell, nucleus and mitochondria by the process of endocytosis. The results obtained from SEM and TEM imaging revealed the attachment of ZnO-NPs on root surface and cellular membranes including tonoplast. Indeed, the cells treated with ZnO-NPs (1000 μg/ml) exhibited an influx of ZnO-NPs in cytoplasm and attachment to the vacuoles (panel E), infiltration of ZnO-NPs onto the nuclear membrane and intracellular junctions with sequestration of ZnO-NPs on nuclear membrane (panel F, G), and degeneration of nuclear constituents and significant swelling of mitochondria (panel F).

### ZnO-NPs induced ROS generation and changes in ΔΨm

**Figure 5:**
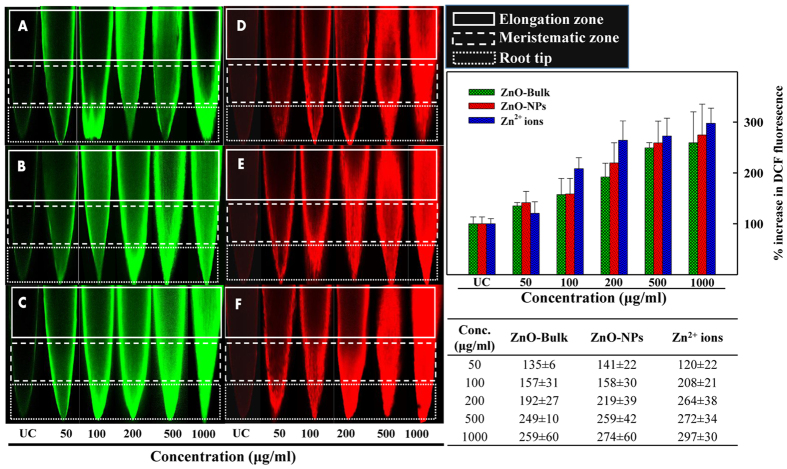
CLSM images of intracellular ROS generation and dissipation of mitochondrial membrane potential in *A. cepa* root after exposure with ZnO-NPs. Panels show the untreated control (UC) and treated roots with varying concentrations of ZnO-Bulk (panel **A**), ZnO-NPs (panel **B**) and Zn^2+^ (panel **C**) stained with 25 μM DCFH-DA. Corresponding bar diagrams represent the % increase in DCF fluorescence of root meristem cells in UC and treated with ZnO-NPs, ZnO-Bulk, and Zn^2+^ ions. CLSM images show the changes in mitochondrial membrane potential (ΔΨm) in *A. cepa* UC roots and those treated with varying concentrations of ZnO-Bulk (panel **D**), ZnO-NPs (panel **E**) and Zinc ions (panel **F**) after staining with Rhodamine123 (1 μg/ml). Dotted, dashed, and solid white color rectangular outlines denote the root elongation zone, meristematic region, and root tip, respectively.

The effects of ZnO-NPs, ZnO-Bulk, and Zn^2+^ ions on ROS production and changes in mitochondrial potential in untreated and treated roots of *A. cepa* were qualitatively measured by histochemical staining with DCFH-DA (25 μM) and Rhodamine 123 (1 μg/ml) using CLS microscope. Results showed a gradual and sharp increase in dichlorofluoresecein (DCF) fluorescence as a result of ROS generation in roots in a concentration dependent manner as compared to untreated control (Fig. 5A–C). Root meristematic zone of *A. cepa* roots was profoundly affected by ROS production. The DCFH-DA is non-fluorescent compound and converts to highly fluorescent DCF when oxidized by intracellular ROS as a result of stress generated by ZnO-NPs, ZnO-Bulk, and Zn^2+^ ions in the vicinity of *A. cepa* roots. DCF fluorescence gives the information about the total oxidative stress of the cell^36^.

Oxidative stress induced by ROS was quantified and significant impact of ZnO-NPs on ROS generation was observed as the NPs attached to the cell surface and internalized. The extent of the increased ROS was highest amongst the roots treated with zinc ions. Results indicate an enhancement in the values of DCF fluorescence as 35, 57, 92, 149, and 159% increase for ZnO-Bulk, 41, 58, 119, 159, and 174% increase for ZnO-NPs, and 20, 108, 164, 172, 197% increase for Zn^2+^ ions upon exposure to 50–1000 μg/ml concentration range (Fig. 5). Compared to the Rh123 fluorescence in control root, the root treated with ZnO-NPs, ZnO-Bulk, and Zn^2+^ ions inevitably show an enhancement in fluorescence of Rh123 at higher concentrations (panels D–F), possibly due to diffusion into the cytoplasm of cells (Fig. 5D–F). The results also demonstrated an upward increase in the fluorescence of Rh123 from root tip to the root elongation zone as compared to untreated control, where no change in *ΔΨm* of cells has occurred.

### FT-IR analysis of root tips

**Figure 6:**
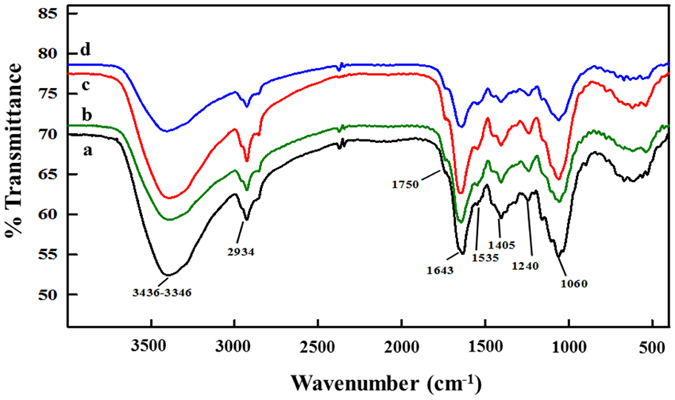
Assessment of *in vivo* interactions of ZnO-NPs, ZnO-Bulk and Zn^2+^ ions with *A. cepa* chemical constituents by FTIR analysis. FTIR spectra of dry root tip powder obtained from untreated roots and those treated under hydroponic conditions in ddw for 12 h, are shown as (**a**) untreated control, and (**b–d**) as roots grown in presence of 100 μg/ml of ZnO-Bulk, ZnO-NPs and Zn^2+^ ions, respectively.

Fine powder of dried roots of *A. cepa* grown in absence and presence of ZnO-NPs, ZnO-bulk, and Zn^2+^ ions at 1000 μg/ml were analyzed to investigate the changes in characteristic signal of biochemical components (Fig. 6). The peaks in the spectra were assigned their corresponding characteristic to organic molecules. Broad peak at 3436–3346 cm^−1^ was assigned to N-H and O-H stretching of proteins and polysaccharides/water^37^,^38^. The peak at 2934 cm^−1^ was due to C-H asymmetric stretch of methylene group of lipids^39^, while peak at 1750 cm^−1^ was due to C=C vibrations of lipids and fatty acids^40^. Peaks located at 1643 and 1535 cm^−1^ were assigned to amide I of α-helix and amide II of β-sheet (C=N, C=C stretching) of secondary structure of proteins^37^,^39^,^40^. The peak positioned at 1405 cm^−1^ was assigned to C-O of COO^−^ groups^31^,^34^. Peaks located at 1240 and 1060 cm^−1^ were due to asymmetric and symmetric P=O stretching of phosphodiesters and stretching of C-O of ribose sugars, respectively^40^.

## Discussion

Recent upsurge in manufactured nanomaterials and their entry into the environment through accidental release during manufacture or via transport through atmospheric emissions, agricultural and domestic effluents, most likely, alters the nature and magnitude of environmental exposure. The impact of such an exposure on terrestrial or aquatic plants has not been widely and systematically explored. Therefore, we have studied the effect of ZnO-NPs (25.5 nm) on *A. cepa* root tip cells at five different concentrations (50, 100, 200, 500, and 1000 μg/ml) with the aim to investigate the phytotoxic and oxidative stress, based on end points, such as root growth inhibition, intracellular ROS generation, dissipation of mitochondrial membrane potential, lipid peroxidation, mitotic index and chromosomal aberrations viz. disturbed metaphase, sticky chromosome, and breaks. Our study distinctly demonstrated the dose dependent inhibition of *A. cepa* root length and root cell viability upon exposure to ZnO-NPs under *in vitro* conditions, which clearly suggests induced phytotoxicity and oxidative stress at cellular level. The results of root growth suppression assay corroborate earlier studies on the inhibition of root growth by ZnO-NPs in corn and cucumber plants^41^. Also, Kouhi *et al*.^20^ demonstrated the phytotoxicity of ZnO-NPs, ZnO-microparticles, and Zn^2+^ ions in terms of root length inhibition of *Brassica napus* seedlings, at the concentration range of 50–500 μg/ml, in the following order as Zn^2+^ > ZnO-MPs > ZnO-NPs, which supports our observations.

In *A. cepa*, we observed more pronounced inhibition with Zn^2+^ ions vis-à-vis ZnO-NPs or bulk ZnO. The Zn^2+^ ions have also been found to be more toxic in ryegrass species compared to ZnO-NPs^42^. It has been suggested that the toxicity of ZnO-NPs may not alone be attributed to their dissolution at the root surface, but also inside the tissue^43^. Depending on the plant species and the experimental milieu, the plausible factors that help promote ZnO-NPs entry into root cells and inhibit seedling growth may be ZnO uptake (external efficiency) or ZnO utilization (internal efficiency) in cells^43^. Since, the greater surface area-to mass ratio of the NPs compared to the bulk metals contributes higher reactivities^43^,^44^, the ZnO-NPs may form a complex with membrane transporters or root exudates before being transported into the plants. Once the NPs enter the plant cells, they may be transported either apoplastically or symplastically from one cell to another via plasmodesmata^42^. The SEM and TEM images of ZnO-NPs treated root cells in our study revealed clear incursion and attachment of ZnO-NPs on the outer and inner surface of cell and nuclear membrane and intracellular cell junctions. Interaction of ZnO-NPs with root surface induced significant morphological changes such as fissures, fractures and spikes and eventually caused damage to mitochondria and nucleus upon internalization. Most likely, the ZnO-NPs could find their way inside the cell, nucleus and mitochondria by the process of endocytosis^42^. The TEM micrographs of this study have revalidated the study of Lin and Xing (2008) regarding the free entry of ZnO-NPs in size range of 9–37 nm, average size 19 ± 7 nm on ryegrass and demonstrated their transportation through plasmodesmata^42^. The data revealed an influx of ZnO-NPs in cytoplasm and attachment to the tonoplast of vacuoles, infiltration to intracellular junctions with sequestration of ZnO-NPs on nuclear membrane, degeneration of nuclear constituents and significant swelling of mitochondria. Significant sequestration of ZnO-NPs in the cytoplasm was noted, probably due to the intracellular matrix (Fig. 4, Panel E, G, and H). It is also suggested that ZnO-NPs <10 nm as demonstrated in Fig. 4 (panel H) can pass the cellular membranes easily and form agglomerates with other molecules within the cell.

ZnO-NPs treatment to roots has induced significant ROS generation and changes in Δ*Ψm*. Histochemical staining with DCFH-DA (25 μM) and Rhodamine 123 (1 μg/ml) using CLS microscope revealed concentration dependent ROS generation in meristematic zone of *A. cepa* roots. The data revealed an upward increase in the fluorescence of Rh123 from root tip to the root elongation zone as compared to untreated control, where no change in Δ*Ψm* of cells has occurred. Faisal *et al*.^35^ have also reported a biphasic change in fluorescence intensity with a marked reduction in fluorescence of Rh123 in tomato root cells at lower NiO-NPs concentrations followed by fluorescence enhancement through diffusion of Rh123 fluorescence. The possible reason for fluorescence intensity reduction at lower concentrations could be due to dissipation of Δ*Ψm* by disruption of proton-moving force and/or the inner membrane permeability. Thus, the relation between greater ROS generation and decline in Δ*Ψm* supports the earlier findings of Liu *et al*.^45^ with water soluble fullerenes. On the contrary, an increase in fluorescence at higher treatment doses relates with the inherent property of mitochondria to swell or shrink in response to changes in Δ*Ψm*. Thus, the intensity of Rh123 fluorescence changes with the morphological transitions^46^. Altered mitochondria can no longer retain Rh123, which leaks out from the mitochondrial membrane into the cytoplasm mainly due to swelling^43^. In *A. cepa* cells, mitochondrial swelling occurs at greater exposure doses of ZnO-NPs, ZnO-Bulk, and Zn^2+^ ions, leading to leakage of Rh123 to cytosolic components of the treated cells and causing hyperpolarization of the dye. The ROS generation within mitochondria occurs in complex I and II of electron transport system through electron leakage, which can directly interact with mitochondrial proteins and lipids, and eventually cause dysfunction^36^. In plants, dissipation of Δ*Ψm* is reported to be an early marker or an essential event in plant apoptosis under stressed conditions^48^. ROS generation by ZnO-NPs, ZnO-Bulk particles may be principally related to their physical interaction with roots and/or dissolution of Zn^2+^ ions^20^. In our study, dose dependent alteration of mitochondrial membrane potential (Δ*Ψm*) was also observed. Since, mitochondria balance the cellular redox potential, interactions between the NPs and mitochondrial membrane, may cause the uncoupling of the respiration, thereby, increasing oxidative stress in the cell^49^,^50^. Mitochondria are well known organelles responsible for ROS generation in cell^51^. Therefore, it is envisaged that interactions of ZnO-NPs, ZnO-Bulk, and Zn^2+^ with electron transport system of mitochondria may lead to the alterations in Δ*Ψm*, which ultimately cause a consequent increase in the ROS level of cell in a dose related fashion. The ROS inducing potential was found to be in the order as Zn^2+^ ions > ZnO-NPs > ZnO-bulk. Indeed, the toxic effect of ZnO-NPs, ZnO-Bulk and Zn^2+^ may also be due to the production of ROS and its interaction with the fatty acids present in lipid membranes, which may cause the formation of lipid peroxides thereby destabilizing the membranes^17^.

Besides, the intracellular ROS mediated conversion of fatty acids, present in biological lipid membranes, to lipid peroxides generates thiobarbituric acid reactive species (TBARS). Our results exhibited the formation of TBARS with ROS at all concentrations of ZnO-NPs and correlates well with alterations in Δ*Ψm*. Significant change in Δ*Ψm* was noted in ZnO-NPs treated roots as compared to ZnO-bulk. Increased levels of TBARS upon treatment of roots with ZnO-NPs, ZnO-Bulk, and Zn^2+^ ions suggested significant lipid peroxidation. Our results support the observations of Kumari *et al*.^27^, who have also reported higher levels of TBARS in case of ZnO-NPs treatment vis-à-vis bulk particles. Some earlier studies have also reported the oxidative stress generation and changes in Δ*Ψm* in germinating seedlings of *Vigna radiata* by Ag-NPs^52^ and *Lycopersicon esculentum* by NiO-NPs^35^. Hernandez *et al*.^53^ detected the oxidative stress generation in *Brassica oleracea* roots under short and long term salt stress by measuring the fluorescence of DCF. Tan *et al*.^54^ have also reported the generation of oxidative stress by MWCNTs in rice seedlings. To combat with the ROS generated under stressed conditions, plants possess antioxidative enzyme defense system, which includes the enzymes such as catalase (CAT), guaiacol peroxidase (GPX) and superoxide dismutase (SOD). The data revealed a gradual increase in % CAT and GPX activity in root cells treated with ZnO-NPs, ZnO-bulk, and Zn^2+^ ions in concentration range of 50–1000 μg/ml. Hernandes-Viezcas *et al*.^55^ support our findings of increase in CAT activity in a dose related manner, in their study on *Prosopisjuliflora velutina* plant treated with ZnO-NPs.

FT-IR spectrum of untreated roots exhibited a variety of sharp peaks, indicative of the pattern of major groups of biochemical constituents of onion root tissue, which corresponds with the FT-IR spectrum reported by Siva *et al*.^56^. A minor shift in the position of peaks located at 1643 and 1535 cm^−1^ and an increase in the % transmittance of treated groups clearly indicates the disturbance in α-helices and β-sheets of secondary structure of root proteins. Similarly, a decrease in the absorbance and shift of the peaks positioned at 1240 and 1060 cm^−1^ supports the damage of phosphate backbone and C-O bond between ribose/deoxyribose sugars of nucleic acids (DNA and RNA) as compared to untreated control. Peak shifting and decrease in the absorbance, in such a way suggests the concentration dependent damage of polysaccharides (3346 cm^−1^) and lipids (1750 cm^−1^) by ZnO-NPs, ZnO-bulk and Zn^2+^ ions. The FT-IR results indicate that ZnO-NPs can adhere to the surface of root cells, subsequently enter into cells and interact with biological macromolecules including DNA, RNA, proteins, lipids and other cellular biomolecules. The quantitative and detailed chemical state information regarding the surface of ZnO-NPs can be further obtained using synchrotron/X-ray photoelectron spectroscopy (XPS) analysis. Quantification of intracellular zinc in digested untreated and treated root tissues by atomic absorption analysis revealed an average of 1.5% internalization of zinc in root cells exposed to 100 μg/ml of ZnO-NPs, compared to 0.041% in untreated control group (results not shown), which explicitly demonstrated the adsorption and internalization of zinc in *A. cepa* root cells, as also validated by SEM and TEM analyses.

Indeed, the standard phytotoxicity tests such as germination and root elongation used in most of the phytotoxicity studies may not be sufficient and sensitive enough for evaluation NPs toxicity in terrestrial plants. Therefore, the cytotoxic and genotoxic potential of NPs was further assessed by studying chromosome alterations. The results demonstrated significant aberrations in chromosomes like disturbed metaphase, sticky chromosome, cell wall disintegration, breaks and decrease in mitotic index, in *A. cepa* root tip cells treated with 50, 100, 200, 500 and 1000 μg/ml of ZnO-NPs. No chromosomal aberrations were observed in the control (untreated onion root tips) and the mitotic index (MI) value was determined to be 60.3%. However, with increasing concentration of the NPs, a substantial decrease in the mitotic index (60.30% to 27.62%) was observed, which corroborate the earlier studies^57^,^58^. The reduction in MI (%) and increase in chromosomal anomalies (%) might be due to the interference of ZnO-NPs and Zn^2+^ ions in suspension with normal DNA replication process and thereby blocking the DNA synthesis at G1 stage. They may also cause chromosomal adherence which ultimately trigger cell death^59^. Significant number of chromosomal breaks, bridges, and stickiness in roots treated with ZnO-NPs, ZnO-Bulk and Zn^2+^ may be either due to the inversion of chromosomal segments or due to the breakage and fusion of chromosomes. Moreover, chromosomal adherence could be due to inaccurate packing of chromosomal fibers within chromosomes that can lead to the inter-connections between chromoproteins that help the chromosomes to connect through sub-chromatidic bridges^60^. Multipolar anaphase has been reported as a consequence of ZnO-NPs or Zn^2+^ interaction with mitotic spindle, which leads to the improper distribution of chromosomes^61^. It has been reported that chromosomal disorientation at metaphase may occur under the stress of ZnO-NPs and Zn^2+^ ions, which might be due to the loss of ability of chromosomes to align at equatorial plate which suggests interaction of ZnO-NPs with the microtubules of spindle fibers^61^. Similar aberrations (chromosomal breaks, stickiness, diagonal anaphase, multipolar anaphase, C-mitosis, and micronucleus) have been reported under the exposure of other NPs (Al_2_O_3_, MWCNT, TiO_2_)^24^,^30^,^31^.

**Figure 7:**
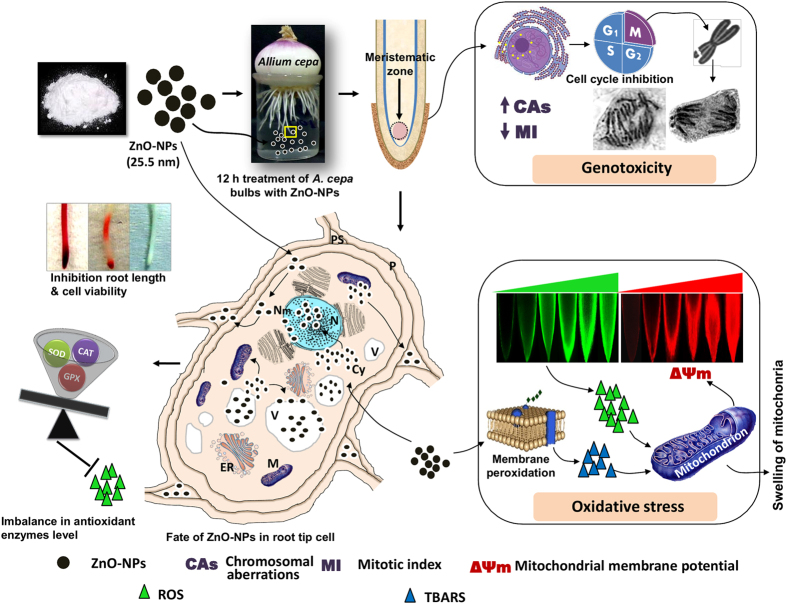
Schematic representation of plausible mechanism of ZnO-NPs interaction with cellular components and induced oxidative stress, cytotoxicity and genotoxicity in *A. cepa* roots. Abbreviations are as follows: N: Nucleus, V: Vacuole, Nm: Nuclear membrane, Cy: Cytoplasm, M: Mitochondria, ER: Endoplasmic reticulum, P:Plasmodesmata, and PS: periplasmic space.

The schematic representation of plausible mechanism of ZnO-NPs behavior in cellular environment is depicted in Fig. 7. It can be speculated that ZnO-NPs when come in close proximity to *A. cepa* roots get deposited by process of adsorption on its surface because the surface forces of root, as adsorbent, are unsaturated. It occurs when both repulsive and attractive forces become balanced. The uptake of NPs across the cell wall largely depends on the size of the particles and fairly constant size of pores across the cell wall^19^. The Fig. 7 reveals the infiltration of ZnO-NPs inside the cell and their attachment to the tonoplast of vacuoles, translocation to intracellular junctions with sequestration of ZnO-NPs on nuclear membrane, degeneration of nuclear constituents and appearance of swollen mitochondria. ZnO-NPs mediated increase in the level of TBARS as a result of membrane lipid peroxidation and generation of intracellular ROS are responsible for the change in mitochondrial ΔΨm coupled with mitochondrial swelling. Even at low concentrations, ZnO-NPs effectively collapse the mitochondrial membrane potential and thus account for the damaging effects of mitochondrial energetics^62^. Swelling of mitochondria with decreased matrix electron density is evident from the study performed with the isolated mitochondria and corroborates our findings^69^. Scheme in Fig. 7 also illustrates the adherence of ZnO-NPs on chromosomes involved in the normal process of mitosis and anomalies caused by them which halt the normal cell division and thus arrest the cell cycle.

## Conclusion

The results ascertained *A. cepa* plants as a bio-indicator for efficient evaluation of cytogenetic effects of metal oxide NPs, specifically ZnO-NPs and Zn^2+^ ions. The experimental data revealed that ZnO-NPs can penetrate into plant tissues and generate intracellular ROS, which eventually induces oxidative imbalance leading to mito-depressive and genotoxic effects, following the scheme depicted in Fig. 7. Since, plants are important component of the ecosystem; they cannot be ignored while evaluating the overall toxicological impact of the NPs in the environment. It is envisaged that quantitative assessment of the adverse effects of NPs in both agricultural and of environmental systems is important. Further investigations are warranted to address the issues involving the NPs uptake kinetics, interaction mechanism within cells, and threshold amount that plants can uphold without any signs of phyto-stress.

## Methods

### Characterization of nanoparticles

ZnO-NPs (size <50 nm, Surface area >10.8 m^2^/g) were obtained from Sigma-Aldrich, USA, and were further characterized for size and shape determination based on UV-Visible, Fourier Transform Infrared (FTIR), fluorescence spectroscopy, X-ray diffraction (XRD) analysis, and Scanning and Transmission Electron Microscopy (Supplementary Methods).

### Preparation of ZnO-Bulk, Zn^2+^, ZnO-NPs dispersions

Stock suspension of ZnO-NPs was prepared by direct addition of 25 mg ZnO-NPs in 5 ml double distilled water (ddw) followed by ultrasonication using Branson Digital Sonifier-102C (CE), 200 watt, 20 kHz, Branson Ultrasonics Corporation, Danbury, CT, USA at 30% amplitude for 20 min to break agglomerates. Diluted suspensions at varying ZnO-NPs concentrations of 50, 100, 200, 500, and 1000 μg/ml were immediately prepared. Similarly, samples of ZnO-Bulk and Zn^2+^ ionic solutions at increasing concentrations of 50, 100, 200, 500, and 1000 μg/ml were simultaneously prepared in double distilled water (ddw).

### Treatment to *A. cepa* roots

Healthy onion bulbs were selected and placed after removing their outer scales onto glass beakers (Borosil, India) filled with 10 ml ddw. Prior to use the glass beakers were rinsed with 1 N HCl. It was also ensured that the ring of root primoridia left undamaged prior to growing in dark^63^. The test was performed at a relatively constant temperature 20 ± 2 °C. Bulbs were provided fresh ddw supply after every 24 h until the roots attain the length of 2–3 cm. Roots were then exposed to treatments with the ZnO-NPs, ZnO-Bulk and Zn^2+^ ions solutions (50, 100, 200, 500, and 1000 μg/ml) under identical conditions for 12 h. Germinated seedlings were protected from direct light to avoid any disturbances in physiology of roots, thereby, minimizing the interruption of cell cycle.

### Measurement of growth

Growth of the bulbs exposed to ZnO-NPs, ZnO-Bulk, and Zn^2+^ ions solutions was measured in terms of their root length. For this, at the end of treatment, experiment was terminated and five roots for each treatment were cut from disc of root primoridia and length was measured individually^63^. Index of tolerance was calculated using following formula:


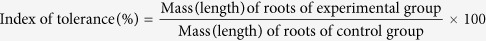


### Optical microscopic analysis of mitosis and scoring of slides

At the end of experiment, roots of onion bulbs exposed to varying concentrations of ZnO-NPs, ZnO-Bulk, and Zn^2+^ solutions were harvested and fixed in a classical plant fixative FAA (Formaldehyde: Alcohol: Acetic acid) in a proportion of 10%:50%:5% plus 35% water and stored at 4 °C until use. Ten root tips per bulb were excised and processed using the method described by Rajeshwari *et al*.^24^. Three best preparations were visualized and scored for mitosis analysis by use of Olympus trinocular microscope (BX60), Japan equipped with ExwaveHAD color video camera (Sony, Japan). 1000 cells were counted per slide. Percent mitotic index was calculated for each treatment using following formula: % Mitotic Index (MI) = TDC/TC × 100, where, TDC is Total number of dividing cells and TC is total number of cells counted.

### Confocal Laser Scanning Microscopic (CLSM) analysis of root cells

Untreated and treated *A. cepa* root tips were taken and incubated with 1 N HCl at 80 °C for 15 min for optimum hydrolysis of cell wall followed by three subsequent washings with ddw. Root tips were then stained with nucleic acid specific stain acridine orange (AO) at 10 μg/ml in phosphate buffer, and incubated for 10 min. Throughout the incubation, dark condition was maintained. Root tip slides were prepared following the method described above. The chromosomal aberrations were then visualized using Leica TCS SPE, confocal laser scanning microscope (Leica Microsystems, Germany).

### Scanning Electron Microscopy (SEM) of treated roots

Apical parts of the roots grown in absence and presence of ZnO-NPs were fixed in 2.5% glutaraldehyde and 2% paraformaldehyde in 0.1 M sodium phosphate buffer (pH 7.2) for 12 h at 4 °C with intermittent stirring. Samples were dehydrated through a graded series of ethanol (10%, 20%, 30%, 50% and 70% once for10 min at each step). Roots were then transferred to critical point dryer using CO_2_ as transitional fluid, which removes water from the specimen and avoids unwanted damage due to the surface tension generated by a liquid/gas interface. Dehydrated roots were then put on the two sided carbon tape fix on SEM stub and visualized under JSM 6510LV scanning electron microscope (JEOL, Tokyo, Japan) at an accelerating voltage of 10 kV.

### Transmission Electron Microscopy (TEM) of treated roots

Small root tips (2 × 2 × 2 mm in size) from untreated and treated with ZnO-NPs were fixed in primary fixative mentioned above and processed following the method of Ali *et al*.^64^.

### Detection of root cell viability (TTC assay)

Metabolically active and inactive root cells under the exposure of test species were detected using the method of Shaymurat *et al*.^65^. The test was performed in dark due the instability of TTC to light. After the incubation, roots were rinsed with ddw and cell viability was observed.

### Effect of ZnO-NPs on ROS production and mitochondrial membrane potential (ΔΨm)

A qualitative *in vivo* method was used to assess the ROS inducing potential of ZnO-NPs and their impact on Δ*Ψm*. For determination of ROS generation, roots from untreated and treated sets were stained with 0.25 μM of 2′, 7′-dichlorofluorescein diacetate (DCFH-DA) for 15 min at 25 °C^27^. Changes in Δ*Ψm* were observed by staining the roots from untreated and treated sets with Rhodamine123 (Rh123) for 20 min^35^. Dark conditions were maintained throughout the process. Stained roots were washed with 0.1 M phosphate buffer to remove the excess stain and then mounted on glass slides. Slides were visualized using Leica TCS SPE, confocal laser scanning microscope (Leica Microsystems, Germany). Images were taken using attached camera component Leica DFC 365 FX Digital Camera. Oxidative stress generated by ROS was also quantified. Briefly, apical part of 10 roots (~5mm) grown in the absence and presence of ZnO-NPs, ZnO-Bulk, and Zn^2+^ in the concentration range of 50–1000 μg/ml were homogenized in 5 ml (0.1 M) phosphate buffer (pH 7.2). Homogenates were centrifuged at 8000 rpm for 15 min. Supernatants were added with DCFH-DA (25 μM) and incubated for 30 min in dark. After incubation, fluorescence intensity of dichlorofluoresecein (DCF) was recorded at the excitation/emission wavelength of 460/530 nm by using spectrofluorophotometer (RF-5301PC), Shimadzu Scientific Instruments, Kyoto, Japan equipped with Xenon lamp (150 W) and RF 530XPC.

### Determination of lipid peroxidation

Determination of lipid peroxidation in treated and untreated roots was performed by measuring malondialdehyde (MDA) following the method of Ohkawa *et al*.^66^. Absorbance was recorded at 450, 532, and 600 nm. MDA level was calculated using the following formula: C (μmol/L) = 6.45 × (Abs_532_ − Abs_600_) − 0.56 × Abs_450._

### Determination of antioxidant enzymes

To evaluate the antioxidant enzyme activities in onion root tissues, the root tips (200 mg) exposed to ZnO-NPs, ZnO-Bulk and Zn^2+^ ionic solutions were crushed with chilled 50 mM phosphate buffer (pH 7.4), using mortar and pestle, followed by the centrifugation at 10,000 rpm for 30 min. Final volume of supernatant was stored at 4 °C until use. Superoxide dismutase (SOD) level was determined using the method of Rajeshwari *et al*.^24^. Guaiacol peroxidase (GPX), and catalase (CAT) activities were assayed following the methods described by Zhang *et al*.^67^ with some modifications. Activity of SOD, GPX and CAT was expressed in terms of percent increase.

### FT-IR analysis of root tips

Root tips (n = 20) each from plants treated with 100 μg/ml of ZnO-NPs, ZnO-Bulk and Zn^2+^ solution and untreated controls were dried in a hot-air sterilizer, York scientific industries Pvt. Ltd. at 50 °C for 48 h. Dried roots were powdered using mortar and pestle and sieved through a mesh. KBr discs were made with IR grade KBr in a mortar in the ratio of 1:100 (sample: KBr). FT-IR analysis was performed in attenuated total reflectance (ATR) mode in the range of 4000–400 cm^−1^ using Spectrum Two FT-IR spectrometer (Perkin Elmer, UK).

### Quantification of zinc in *A. cepa* roots by AAS

The internalization of ZnO-NPs, ZnO-Bulk and Zn^2+^ ions in *A. cepa* root tip cells was determined using a double beam atomic absorption spectrophotometer model: GBC 932B plus, Australia (Supplementary Methods).

### Statistical analysis

Data represent the mean ± S.D. of two independent experiments in triplicate. Statistical analysis was performed using one-way analysis of variance (ANOVA) and Tukey’s test, using Minitab 17.

## Additional Information

**How to cite this article**: Ahmed, B. *et al*. Mitochondrial and Chromosomal Damage Induced by Oxidative Stress in Zn^2+^ Ions, ZnO-Bulk and ZnO-NPs treated *Allium cepa* roots. *Sci. Rep.* **7**, 40685; doi: 10.1038/srep40685 (2017).

**Publisher's note:** Springer Nature remains neutral with regard to jurisdictional claims in published maps and institutional affiliations.

## Supplementary Material

Supplementary Information

## Figures and Tables

**Table 1:** Chromosomal aberrations and mitotic index (MI) in ZnO-Bulk, ZnO-NPs, and Zn^2+^ ions treated *Allium cepa* root meristem cells.

Conc. (μg/ml)	ZnO-Bulk Chromosomal aberrations (%)					ZnO-NPs Chromosomal aberrations (%)					Zn^2+^ ions Chromosomal aberrations (%)				
	CS	CB(s)	DA	IM	MI (%)	CS	CB(s)	DA	IM	MI (%)	CS	CB(s)	DA	IM	MI (%)
0	—	—	—	—	58.4 ± 1.8	—	—	—	—	58.4 ± 1.8	—	—	—	—	58.4 ± 1.8
50	0.86 ± 0.35	0.83 ± 0.25	0.76 ± 0.1	0.8 ± 0.1	57.4 ± 1.86	2.03 ± 0.2	1.9 ± 0.1	1.6 ± 0.4	2.33 ± 0.6	53.06 ± 0.9	5.56 ± 0.4	4.53 ± 0.4	2.33 ± 0.2	3.2 ± 0.26	56.3 ± 2.25
100	1.63 ± 0.3	0.96 ± 0.3	0.96 ± 0.3	1.16 ± 0.41	53.4 ± 1.91	2.43 ± 0.3	2 ± 0.1	1.93 ± 0.2	2.66 ± 0.2	46.46 ± 2	6.43 ± 0.4	5.1 ± 0.1	4.46 ± 0.4	4.36 ± 0.3	45.3 ± 4.65
200	1.8 ± 0.5	1.63 ± 0.41	1.86 ± 0.15	1.46 ± 0.6	49.0 ± 0.97	3.2 ± 0.26	2.93 ± 0.15	3.16 ± 0.94	3.16 ± 0.2	42.6 ± 1.5	7.26 ± 0.2	9.23 ± 0.2	6.4 ± 0.45	5.46 ± 0.5	40.7 ± 1.67
500	3.2 ± 1.08	2.9 ± 0.1	3 ± 0.55	3.16 ± 0.3	44.6 ± 1.04	4.3 ± 0.45	4.66 ± 0.41	3.96 ± 0.15	4.03 ± 0.9	37.16 ± 1.3	9.93 ± 0.2	9.6 ± 0.3	6.26 ± 0.5	5.43 ± 0.4	34.6 ± 2.65
1000	3.7 ± 0.52	3.3 ± 0.26	2.8 ± 0.43	3.3 ± 0.7	37.5 ± 2.12	5.53 ± 0.6	5.7 ± 0.62	5.46 ± 0.5	4.33 ± 0.5	30.23 ± 0.4	10 ± 0.26	10.2 ± 0.2	5.76 ± 0.4	5.36 ± 0.0	27.7 ± 1.55

CS: Chromosome stickiness; CB(s): Chromosome bridge(s); DA: Disturbed anaphase; IM: Irregular metaphase. For ZnO-Bulk, ZnO-NPs, and Zn^2+^ ion treated sets, a total 1000 cells per slide were analyzed. The data represent the mean ± SD of experiments in triplicate.
