# Supplementary material for: Mitochondrial and Chromosomal Damage Induced by Oxidative Stress in Zn2+ Ions, ZnO-Bulk and ZnO-NPs treated Allium cepa roots
Source: Sci Rep. 2017 Jan 25;7:40685. doi: 10.1038/srep40685 (PMC5264391; doi:10.1038/srep40685)
Supplement: Supplementary Information [file srep40685-s1.pdf]

Mitochondrial and Chromosomal Damage Induced by Oxidative Stress in Zn^{2+} Ions, ZnO-Bulk and ZnO-NPs treated *Allium cepa* roots

Bilal Ahmed[a], Sourabh Dwivedi[a,d], Malik Zainul Abidin[c], Ameer Azam[d], Majed Al-Shaeri[e], Mohammad Saghir Khan[a], Quaiser Saquib[b], Abdulaziz A. Al-Khedhairy[b], *Javed Musarrat[a,f]

[a]Department of Agricultural Microbiology, Faculty of Agricultural Sciences, Aligarh Muslim University, Aligarh-202002, U.P., India

[b] Department of Zoology, College of Science, King Saud University, P.O. Box 2455, Riyadh 11451, Saudi Arabia.

[c]Department of Biotechnology, Jamia Hamdard, New Delhi-110062, India

[d]Department of Applied Physics, Aligarh Muslim University, Aligarh-202002, U.P., India.

[e]Department of Biological Sciences, Faculty of Science King Abdulaziz University, Jeddah, Saudi Arabia.

[f]School of Biosciences and Biodiversity, Baba Ghulam Shah Badshah University, Rajouri, J & K, India.

*Corresponding author:
Prof. Javed Musarrat
Department of Agricultural Microbiology
Faculty of Agricultural Sciences
Aligarh Muslim University
Aligarh 202002, U.P. India
Tel: +919760785651
Email: musarratj1@yahoo.com

Supplementary Information:

Supplementary Figures:

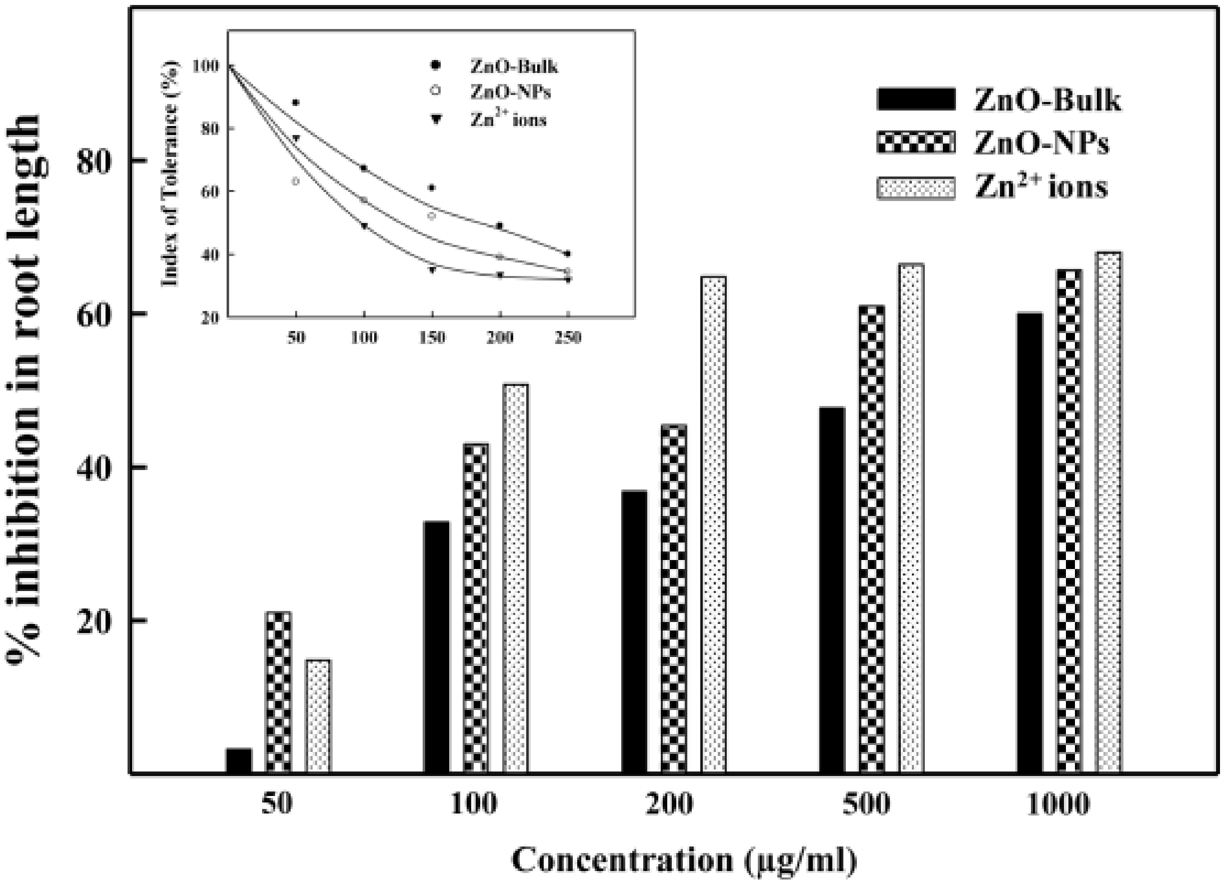

Supplementary figure S1. Percent root length inhibition in *A. cepa* induced by ZnO-NPs, ZnO Bulk and Zn^{2+} ions. Histogram shows the % root length inhibition as a function of concentration in the range from 50-1000 µg/ml, taking separate untreated controls as 100 %, under each treatment condition. Inset shows the index of tolerance (% IT).

Phytotoxic effects of ZnO-NPs, ZnO-bulk, and Zn^{2+} ions were assessed in terms of root length inhibition. The results of root length inhibition as a function of concentration in the range of 50 to 1000 µg/ml are shown in Supplementary Figure S1. Almost 20 % and 14.85 % inhibition occurred at a concentration of 50 µg/ml with ZnO-NPs and Zn^{2+} ions compared to 3% with ZnO-bulk. Root length inhibition at 500 µg/ml has been observed to be 60.94% and 67.9% with ZnO-NPs and Zn^{2+} ions vis-a-vis 47.7 % in ZnO-bulk. However, at the highest concentration of 1000 µg/ml, the extent of % inhibition becomes almost similar i.e. 65.6 %,

67.9% and 60 % with ZnO-NPs, Zn^{2+} ions and ZnO-Bulk, respectively. Index of tolerance as determined based on the algorithm specified in methods section has been found 34% ZnO-NPs, 32% Zn^{2+} ions and 40% ZnO-bulk at the greatest concentration of 1000 µg/ml.

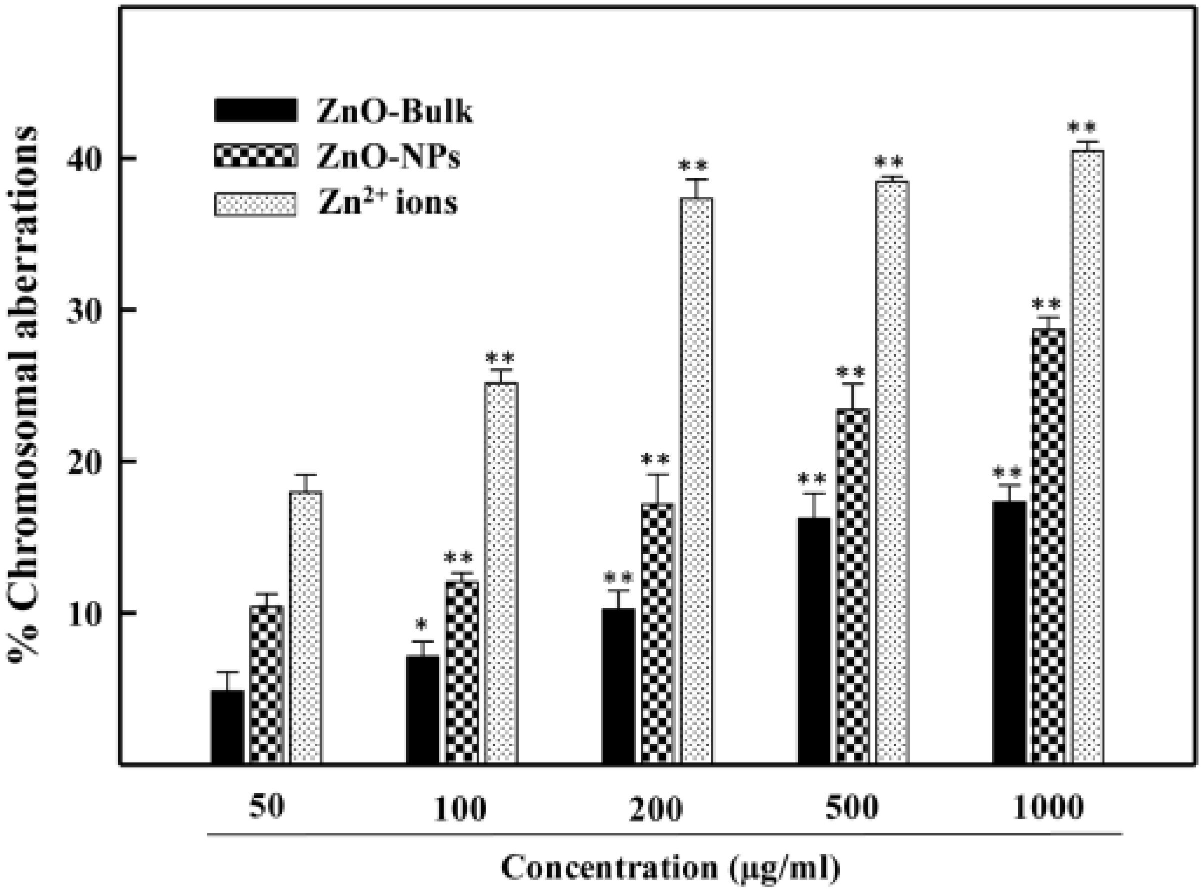

Supplementary figure S2. Extent of chromosomal aberrations in *A. cepa* root cells exposed to ZnO-NPs, ZnO-Bulk, and Zn^{2+} ions in the concentration range of 50-1000 µg/ml. Error bars indicate the mean ± SD of experiments in triplicate (*p<0.01, **p<0.001).

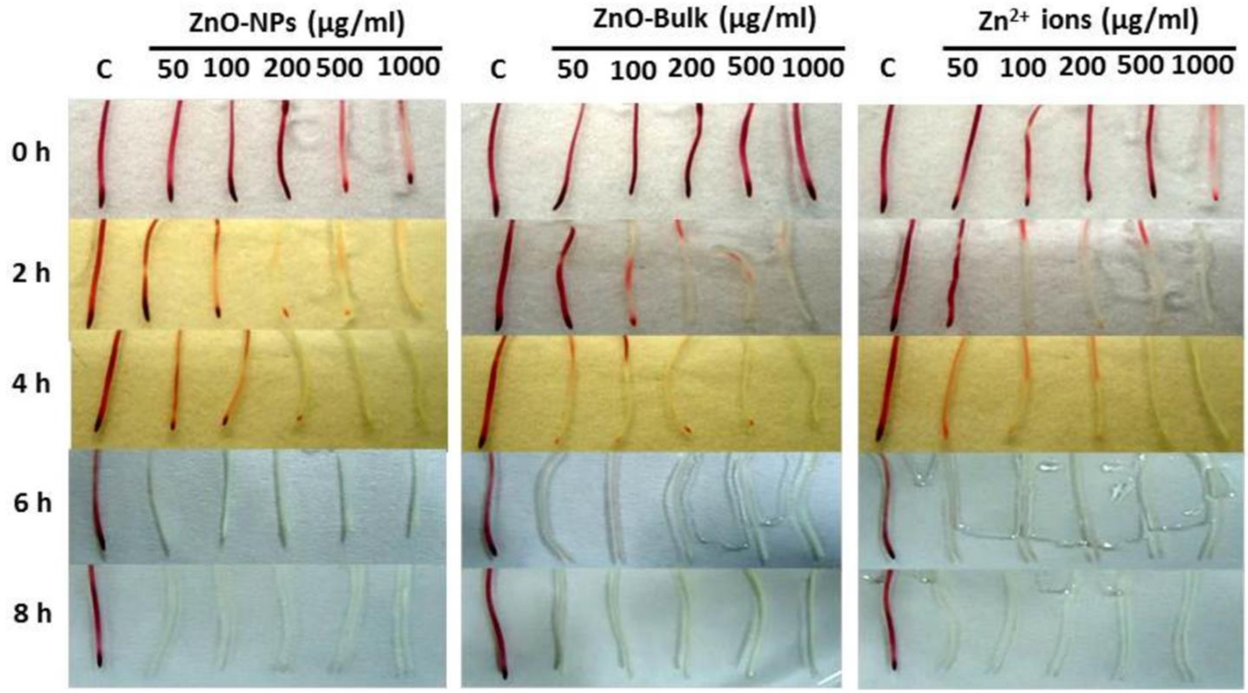

Supplementary figure S3. Qualitative assessment of cell viability by TTC staining of *A. cepa* roots grown in the absence (control) and presence of ZnO-NPs, bulk, and ions in the concentration range from 50-1000 µg/ml, as a function of time of incubation.

Root cell viability was detected by observing a red color compound TPF (1, 3, 5-triphenylformazan) formation in living cells, which is a reduction product of white color TTC (triphenyltetrazolium chloride) due to the activity of cellular dehydrogenases. This test clearly differentiates between metabolically active and inactive cells as TTC remains white in color in the regions where dehydrogenase enzymes are inactive. Cell viability in untreated roots and those treated with increasing concentrations (50-1000 µg/ml) of ZnO-NPs, bulk, and ions was assayed at 2 h interval up to 8 h. Regions of root stained red were considered as viable while regions in white were dead. After 2 h almost all cells treated with 200-1000 µg/ml were observed metabolically inactive except some actively growing cells of root tip while after 8 h, all cells whether from root tip, meristematic zone or elongation zone were found dead except untreated cells (Supplementary Figure S3).

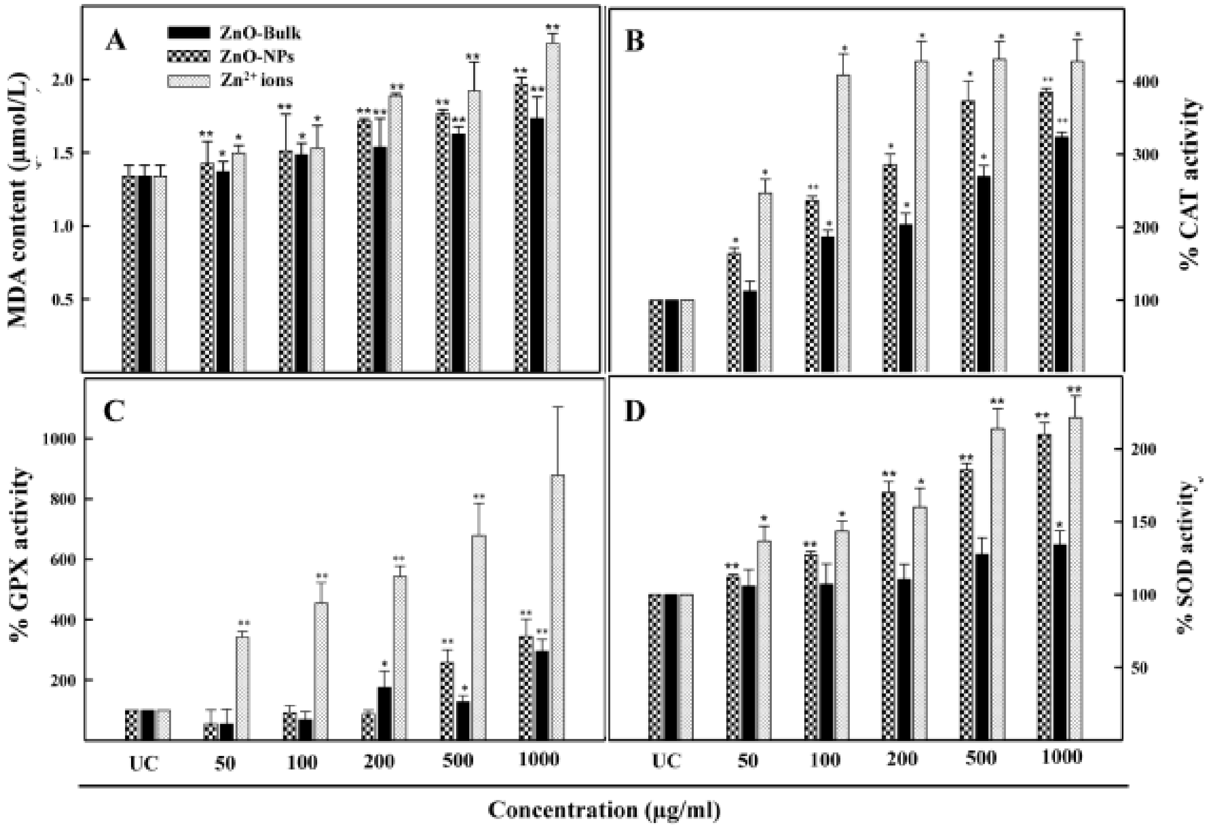

Supplementary figure S4. Effect of ZnO-NPs, ZnO-Bulk, and Zn^{2+} ions on membrane lipid peroxidation (MDA content) (A), CAT (B), GPX (C), and SOD (D) activity in *A. cepa* root meristem cells compared to untreated control (UC). Values from three replicates are expressed as mean ± SD (*p<0.05, **p<0.01).

In order to assess the effects of ZnO-NPs, ZnO-bulk, and Zn^{2+} ions in the concentration range of 50-1000 μg/ml on membrane lipid peroxidation, content of thiobarbituric acid reactive species (TBARS), a byproduct of lipid peroxidation, was measured. TBARS assay measures the malondialdehyde (MDA) content formed due to the decomposition of certain primary and secondary lipid peroxidation products. TBARS concentration for 50, 100, 200, 500, and 1000 μg/ml ZnO-NPs was found to be 1.36 ± 0.14, 1.51 ± 0.25, 1.71 ± 0.01, 1.76 ± 0.02, and 1.96 ± 0.04 μmol/L, respectively compared to 1.34 ± 0.07 μmol/L in untreated control (Supplementary Figure S4 A). For ZnO-bulk, it was 1.36 ± 0.06, 1.48 ± 0.07, 1.53 ± 0.19, 1.62 ± 0.05, and 1.73 ± 0.14 μmol/L under identical conditions. TBARS contents for Zn^{2+} ions were significantly greater with 1.42 ± 0.05, 1.43 ± 0.15, 1.88 ± 0.01, 1.92 ± 0.19, and 2.24 ± 0.06 μmol/L at 50, 100, 200, 500, and 1000 μg/ml, respectively.

Supplementary Figure S4 B shows a gradual increase in % CAT activity of ZnO-NPs, ZnO-Bulk, and Zn^{2+} ions in concentration range of 50-1000 µg/ml. Zn^{2+} ions caused maximum increase at 100 µg/ml with no further enhancement in CAT activity at higher concentrations. However, ZnO-NPs and ZnO-bulk treatment to root cells resulted in concentration dependent increase in CAT activity. In general, the level of CAT, SOD, and POD enzymes was found increased in treated cells in the order as Zn^{2+} ions > ZnO-NPs > ZnO-Bulk. We have assessed the GPX activity of root cells from untreated and treated groups by measuring the amount of tetraguaiacol at 470 nm. It is produced in cells as a result of interaction of guaiacol with H_2O_2 generated under the stress of ZnO-NPs, bulk, and ions. Peroxidase (GPX) activity in roots treated with ZnO-NPs (50-100 µg/ml) was relatively constant and found significantly ($p \leq 0.001$) increased in cells treated with 500 and 100 µg/ml ZnO-NPs. Whereas, the Zn^{2+} ions resulted in concentration (50-100 µg/ml) dependent increase in enzyme activity (Supplementary Figure S4 C). Almost similar trend of increase in activity of enzyme superoxide dismutase (SOD) was observed in the root cells of treated groups as compared to control (Supplementary Figure S4 D).

Supplementary Methods

Characterization of nanoparticles

UV-Visible spectroscopy. ZnO-NPs were characterized by absorbance measurement by use of Cintra10$_e$ double beam UV-Visible spectrophotometer (GBC Scientific Equipments Pvt. Ltd., Australia) in the wavelength range of 250-800 nm at an interval of 1 nm[1].

Fourier Transform Infrared (FTIR) spectroscopy. FT-IR spectra of ZnO-NPs was recorded by use of Perkin Elmer FT-IR spectrometer Spectrum Two (Perkin Elmer Life and Analytical Sciences, CT, USA) in KBr phase. ZnO-NPs were subjected to FTIR measurements in the range of 4000-450 cm^{-1} [2].

Fluorescence measurement. Fluorescence emission spectrum of ZnO-NPs was recorded by use of Shimadzu spectrofluorophotometer (RF-5301PC), Shimadzu Scientific Instruments, Kyoto, Japan, equipped with Xenon lamp (150W) and RF 530XPC instrument control software using a

quartz cell of 1 cm path. The fluorescent intensity was recorded at an excitation (λ_{exc}) of 320nmand emission wavelength at 350-600nm[3].

X-ray diffraction (XRD) analysis. XRD pattern of ZnO-NPs was recorded by use of MiniFlexII Desktop X-ray diffractometer (Rigaku Corporation, Tokyo, Japan) at 30kV/15mA. Peak intensities were observed from 20º to 80º 2θ angles. Average particle size of ZnO-NPs was calculated by Debye-Scherrer's equation: $d=0.94\lambda/\beta\cos\theta$; where, d is the average crystalline size of ZnO-NPs, 0.94 is Scherrer's constant, λ is the wavelength of X-rays (1.541ºA), θ is the Bragg diffraction angle, β is full-width-at-half-maximum (FWHM) of ZnO-plane 101[4].

Scanning and Transmission Electron Microscopy (SEM/TEM). Scanning electron microscopic analysis was done using fine powder of the ZnO-NPs on a carbon tape in JSM 6510LV scanning electron microscope (JEOL, Tokyo, Japan) at an accelerating voltage of 25 kV. Transmission electron microscopy analysis was carried out using JEOL 100/120 kV TEM (JEOL, Tokyo, Japan) operating at a voltage of 200 keV. Samples were prepared by drying 10µl of ultrasonicated ZnO-NPs on a copper grid at room temperature[1].

Quantification of zinc in *A. cepa* roots by AAS. The *A. cepa* roots exposed to various concentrations (50-1000 µg/ml) of all test species were washed with ddw prior to drying at 65°C for 48 h. The dried roots were subsequently ground to fine powder using mortar and pestle and then sieved through 1 mm nylon mesh. The powdered samples (1 g) from each group were digested using the 10 ml mixture of concentrated HNO_3 and $HClO_4$ at the ratio of 3:2 v/v at 80°C for 12 h. The digests were diluted with ddw to 100 ml and filtered through 0.45 µm membrane filter and analyzed by AAS. Each experiment was performed in triplicate and mean absorbance was calculated for each set of treatment. The concentration of zinc ions in roots were calculated by plotting a standard curve and expressed as µg/ml.

Supplementary references

1. Wahab, R. *et al.* ZnO nanoparticles induced oxidative stress and apoptosis in HepG2 and MCF-7 cancer cells and their antibacterial activity. *Colloids and Surfaces B: Biointerfaces*. **117,** 267-76 (2014).
2. Umar, A., Rahman, M. M., Vaseem, M. M. & Hahn, Y. B. Ultra-sensitive cholesterol biosensor based on low-temperature grown ZnO nanoparticles. *Electrochemistry Communications* **11,** 118–121 (2009).
3. Huang, M. H. *et al.* Catalytic Growth of Zinc Oxide Nanowires by Vapor Transport. *Adv. Mater.* **13(2),** 113-116 (2001).
4. Dwivedi, S. *et al.* Reactive Oxygen Species Mediated Bacterial Biofilm Inhibition via Zinc Oxide Nanoparticles and Their Statistical Determination. *PLoS ONE* **9(11),** e111289. doi:10.1371/journal.pone.0111289 (2014).
